# Serum cytokines in childhood-Takayasu arteritis: Are they biomarkers for indolent disease activity?^⁎^

**DOI:** 10.1016/j.clinsp.2023.100256

**Published:** 2023-07-27

**Authors:** Gleice Clemente, Maria Teresa Terreri, Rosa M.R. Pereira, Bruno Gualano, Clovis Artur Silva, Alexandre Wagner de Souza

**Affiliations:** a Pediatric Rheumatology Unit, Department of Pediatrics, Universidade Federal de São Paulo, São Paulo, SP, Brazil; b Division of Rheumatology, Hospital das Clínicas HCFMUSP, Faculdade de Medicina, Universidade de São Paulo, São Paulo, SP, Brazil; c Pediatric Rheumatology Unit, Children and Adolescents Institute, Hospital das Clínicas HCFMUSP, Faculdade de Medicina, Universidade de São Paulo, São Paulo, SP, Brazil; d Division of Rheumatology, Department of Medicine, Universidade Federal de São Paulo, São Paulo, SP, Brazil

Childhood onset-Takayasu Arteritis (c-TA) is a chronic and recurrent disease, where arterial lesions may progress even in patients in apparent disease remission. To date, there is no gold standard for assessing disease activity in c-TA, making clinical evaluation of c-TA patients a challenge.

Research about valid biomarkers for disease activity in TA has been published during the last few decades, however, there are dissimilarities among the results.^1^ Furthermore, c-TA seems to be different than in adults, with a more pronounced inflammation and more systemic symptoms, such as fever and fatigue, at the beginning of the disease.^2^

It is worth noting that there is a paucity of studies assessing disease activity parameters in c-TA. The aim of this study was to assess the levels of serum cytokines in c-TA patients in order to find a valid biomarker for indolent disease in patients in remission by clinical scores.

This is a cross-sectional study, which included c-TA patients from three Brazilian reference centers in Pediatric Rheumatology. Inclusion criteria were a fulfillment of EULAR/PRINTO/PRES c-TA classification criteria and being in clinical remission according to the Indian Takayasu Clinical Activity Score (ITAS) 2010 and Pediatric Vasculitis Activity Score (PVAS).^3^, ^4^, ^5^ Immunosuppressive drugs were withdrawn during two half-lifetime periods before blood collection. Fourteen Healthy Controls (HC), age and sex-matched, were included.

Assessment of the following serum cytokines was performed: Interleukin-1 receptor antagonist (IL-1ra), Interleukin-1 beta (IL-1β), Interleukin-6 (IL-6), Interleukin-10 (IL-10), Interleukin-12p70 (IL-12p70), Tumor Necrosis Factor Alpha (TNF-α), Interferon Gamma (IFN-γ), Vascular Endothelial Growth Factor (VEGF) and Platelet-Derived Growth Factor (PDGF).

Twelve c-TA patients were evaluated (66.7% girls). The mean age was 18.7±2.84 years, and the median time between disease onset and the diagnosis was 11.5 (5.0‒25.5) months. The median follow-up time of c-TA patients was 10 (7.0‒11.8) years. All patients were on medication: 7 (58%) were on biological Disease-Modifying Antirheumatic Drugs (DMARDs) (i.e., 4 on infliximab, 2 on adalimumab, and 1 on tocilizumab) combined with conventional DMARDs.

Serum cytokine levels presented no differences between c-TA patients and HC (p > 0.05) (Table 1). When the extension of the disease was assessed, diffuse arterial involvement (represented by angiographic type V Hata classification) and localized disease (represented by angiographic types I, IIa, and III Hata classification) revealed no differences in cytokine levels.^6^

**Table 1:** Cytokine levels of childhood-onset Takayasu arteritis patients and healthy controls.

Laboratory Markers	Patients (N = 12)	Controls (*N* = 14)	*p*-value
IFN-γ (pg/mL)	2.3 (1.4‒8.7)	1.3 (0.9‒2.2)	0.089
IL-10 (pg/mL)	9.4 (4.5‒13.4)	7.4 (4.5‒10.2)	0.410
IL-12p70 (pg/mL)	3.5 (2.6‒9.6)	4.9 (3.2‒5.2)	0.502
IL-1ra (pg/mL)	41.6 (32.9‒62.3)	39.8 (21.5‒43.7)	0.135
IL1-β (pg/mL)	2.1 (1.6‒3.3)	2.1 (1.9‒2.6)	0.757
IL-6 (pg/mL)	8.0 (3.6‒27.0)	9.2 (2.6‒14.8)	0.607
TNF-α (pg/mL)	13.7 (6.8)	13.8 (6.5)	0.969
VEGF (pg/mL)	1.9 (1.1‒2.6)	1.5 (1.4‒2.0)	0.440
PDGF (pg/mL)	44.2 (31.2‒674.7)	130.8 (53.9‒1010.7)	0.446

IFN, Interferon; IL, Interleukin; N, Number of participants; PDGF, Platelet-Derived Growth Factor; TNF, Tumor Necrosis Factor; VEGF, Vascular Endothelial Growth Factor. Results are presented as mean and standard deviation or as median and interquartile range.

Progression of arterial lesions in TA is observed in a significant number of patients even during immunosuppressive therapy. Therefore, it is of paramount importance to identify surrogate markers indicating smoldering arterial inflammation, despite therapy, in order to escalate treatment.

It is speculated that multiple pathological processes are involved in TA, though a clear pathogenesis has not yet been established. In a previous study from the group using ^18^F-Fluordeoxiglucose ‒ Positron Emission Tomography/Magnetic Resonance Imaging (FDG-PET/MRI) in c-TA, 10 of 12 patients presented high arterial FDG-uptake (visual score = 3), despite undergoing treatment and apparent clinical remission.^7^ This finding reinforces the need to seek out a reliable biomarker that could reflect smoldering disease activity.

In this study, serum cytokine levels were similar between c-TA patients in clinical remission and HC. Of note, the patients had long periods of follow-up and were on long-term immunosuppressants. Studies with adult TA revealed that plasma/serum cytokine levels were not useful to differentiate patients in active disease and in remission, especially when using anti-cytokine therapy.^8^^,^^9^ The same results regarding cytokine levels were found in other studies between TA patients and HC.^10^

In conclusion, the search for reliable biomarkers to identify indolent disease activity is also a challenge in c-TA patients. Further prospective and multicenter studies are needed in order to improve the study of biomarkers involved in indolent vascular inflammation during the follow-up of c-TA patients.

## Compliance with ethical standards

This study was approved by the local Institutional Review Boards. The patients were included only after the signature of their informed consent and assent.

## Declaration of Competing Interest

The authors declare no conflicts of interest.
